# Chemistry for the Warfighter: Midshipman Research at the U.S. Naval Academy

**DOI:** 10.1021/acsomega.5c07144

**Published:** 2025-12-30

**Authors:** Leighanne A. Brammer Basta, Dianne J. Luning Prak, Melonie A. Teichert, Elizabeth A. Yates

**Affiliations:** Department of Chemistry, 32722 United States Naval Academy, 572M Holloway Road, Annapolis, Maryland 21402, United States

## Abstract

The United States Naval Academy (USNA) is an exclusively undergraduate institution that awards every graduate with a Bachelor of Science degree and prepares them for military service in the Navy or Marine Corps. This viewpoint highlights (1) how chemistry is integrated into our rigorous military program of study, (2) how our independent research courses help to prepare future Naval and Marine Corps officers, (3) how midshipmen contribute to publishable military-relevant research, and (4) how external collaborative efforts increase the caliber and impact of research.

## Graphical abstract

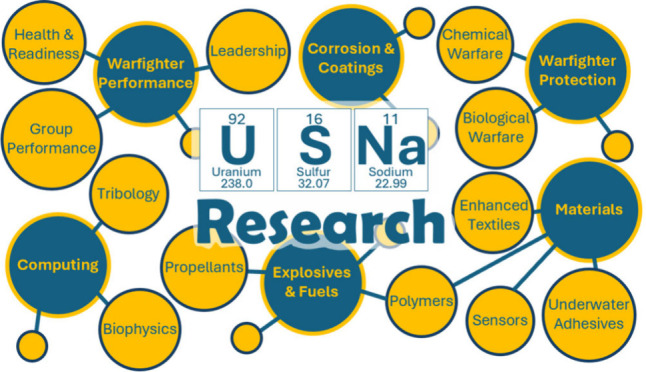

## Introduction

The United States Naval Academy (USNA) is a highly selective, exclusively undergraduate institution with more than 4,400 students (midshipmen) representing all 50 states, some U.S. territories, and a handful of allied countries. Midshipmen are appointed officers-in-training that commission as ensigns or second lieutenants in the Navy or Marine Corps, respectively, and assume careers in naval aviation, surface warfare, the submarine force, special warfare and special operations, cyber- and information warfare, the medical service corps, or Marine Corps specialties. USNA and many of its programs are accredited by nongovernmental organizations that evaluate educational institutions and programs by set standards, including the Middle States Commission on Higher Education (MSCHE) and ABET (previously the Accreditation Board for Engineering and Technology). While two-thirds of our students major in STEM fields, all midshipmen, regardless of their major, graduate with a Bachelor of Science degree because of the technical core-course requirements in chemistry, physics, engineering, and mathematics. In addition to a rigorous academic curriculum that requires midshipmen to attend class, students have robust physical readiness and military requirements. Approximately one-third of our students are Division I athletes (the highest division of intercollegiate athletics sanctioned by the National Collegiate Athletic Association in the United States), and another third play intercollegiate club sports.

USNA maintains a rigorous chemistry program of study, both for a year-long required core course and for an American Chemical Society (ACS)-accredited chemistry degree, with a distinct focus on preparing graduates for military service. Throughout our curriculum and in their research experience, we help midshipmen to (1) learn military applications of chemistry, (2) develop the skills (e.g., decision making, problem-solving, oral and written communication, technical proficiency) and confidence needed to command naval units and lead sailors and marines in missions at sea, on land, or in the air, and (3) learn and master a variety of complex instrumentation, which will translate to adapting to the highly technical arena of modern naval systems.

## The USNA Chemistry Curriculum

Since its foundation in 1845, USNA has placed emphasis on chemistry as a critical area of study in supporting the Academy’s mission of developing and educating the warfighter. Chemistry was included in USNA’s original organization of six professorships.^1^ Over the past 180 years, the chemistry course of study at USNA has transformed from a senior-level course to a required component of the freshmen (plebe) core curriculum as foundational to USNA’s STEM-focused academic program to meet current and future technical needs of the Navy.^2^ The two-semester general chemistry course sequence (Foundations of Chemistry I and II), affectionately referred to as “Plebe Chemistry” by USNA midshipmen and faculty, is designed to provide a strong foundation in underlying chemical principles for naval fleet readiness, to develop scientific reasoning, and to build critical problem-solving and communication skills that are essential across all commissionable warfare communities. A critical difference between plebe chemistry at USNA and foundational chemistry courses at civilian institutions is the additional emphasis of chemistry-centric naval applications (e.g., explosives, corrosion, etc.; see the Supporting Information), which are embedded in lectures, interactive activities,^3^ and laboratory experiments^4^ (Figure 1).

**Figure 1:**
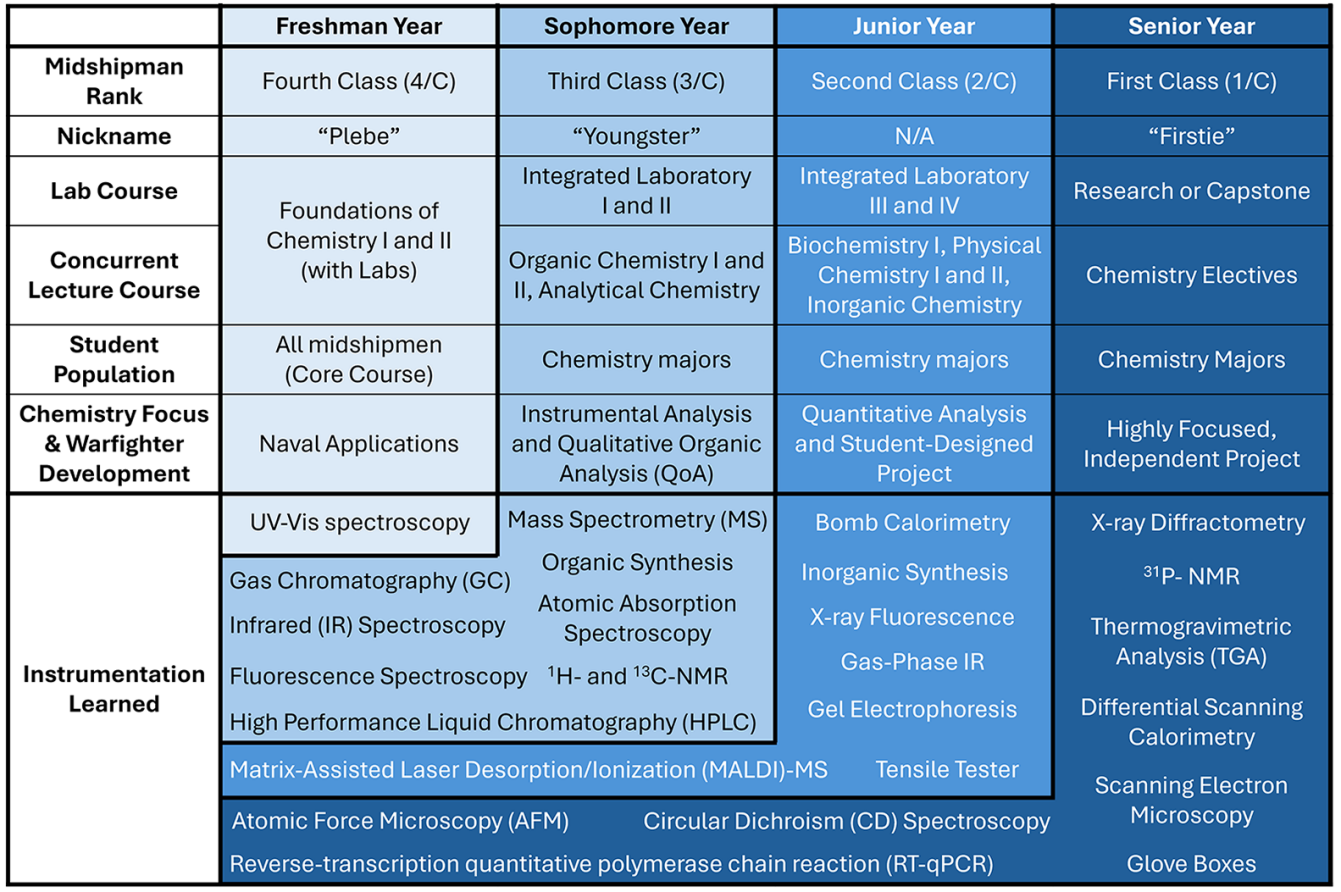
USNA’s core and chemistry major curriculum prepares midshipmen with the technical proficiencies necessary to conduct meaningful independent research and lead sailors and marines as naval officers in the fleet.

Despite 25–40 midshipmen graduating each year with ACS-accredited degrees, no chemistry majors will immediately pursue a career in the chemical sciences following graduation. Instead, our majors join every naval warfare community after commissioning and graduation (e.g., aviation, surface warfare, etc.),^5^ and a small number (∼10 midshipmen) go directly to medical school, from which they will serve as doctors in the Navy Medical Service Corps.^6^ In order to help prepare students for meaningful research experiences during their senior year and for success as future naval officers, USNA adopted a rigorous four-semester Integrated Laboratory (IL) sequence more than 20 years ago that begins in the fall of sophomore year and runs through the spring of junior year (Figure 1).^7,8^ Each semester, midshipmen spend 6 hours per week in IL conducting experiments that investigate multiple areas of chemistry simultaneously, depending on the courses in which they are concurrently enrolled. They are introduced to various means of communication (memoranda, formal reports), laboratories that require critical thinking (Qualitative Organic Analysis and student-designed projects), and highly technical, state-of-the-art instrumentation. A number of experiments are tailored to be relevant to fleet applications (e.g., quantum dots and nanotechnology, superconductors, polymers, etc.; see the Supporting Information). These experiences in the IL sequence instill skills that simultaneously prepare students for independent research in their senior year and for their future military service.

## Midshipman Research Develops Skills Required to Successfully Lead Sailors and Marines in the Fleet

In accordance with the mission of USNA to “promote and maintain an environment in which research and scholarly activities contribute to the professional growth of faculty and the educational growth of midshipmen,”^9^ all USNA chemistry majors are required to participate in research or a Capstone experience.^10^ Undergraduate research is a key component of the student experience highlighted by the ACS’s Committee on Professional Training^11^ and the Council on Undergraduate Research,^12^ enabling students to integrate and reinforce chemistry knowledge and skills, develop scientific literacy and communication competencies, foster collaboration with the broader scientific community, and create new scientific knowledge through close faculty mentorship.^13–15^ More specific to the mission of USNA, undergraduate research develops skills directly applicable to military leadership like adaptability, resilience, and the ability to synthesize data, assess results, and make informed decisions.^16^ Midshipmen graduates are expected to be technically proficient and able to apply problem-solving approaches to advanced naval systems. When reflecting on his undergraduate research experience, a recent graduate wrote, “These projects drive individual ownership, requiring students to develop and execute their own plans, tackle complex problems, and adapt to challenges without a cookie-cutter script. This kind of independent problem-solving is a critical skill in the nuclear pipeline and beyond.”^17^

Faculty and student research is enabled by a well-resourced, state-of-the-art instrument suite. The USNA Chemistry Department houses more than five million dollars of equipment, and midshipmen utilize this instrumentation throughout their four years of study (Figure 1). Midshipmen are also trained in rigorous statistical analysis and technical problem-solving. These skills enable them to adapt to the complex technical sphere of naval warfare in the fleet.

The oral and written communication skills that USNA midshipmen develop are vital to their future service, as described in the Officer Professional Core Competency Manual.^18^ Midshipmen are largely detailed-oriented, and our majors develop skills in proper notebook keeping, oral communication, and memoranda writing. Memoranda are an important means of communication in the fleet, and specific protocols must be followed in military correspondence.^18,19^ Effective, accurate, and ethical communication in both written and oral forms is essential for Navy and Marine Corps officers that lead and manage sailors and marines in order to ensure mission readiness. As part of their research project course, midshipmen prepare poster presentations, reports (fall and spring semesters), and an oral presentation. The spring semester research posters are presented during Capstone Day,^10^ which is attended by military sponsors, governmental laboratories and personnel, and various dignitaries. High quality oral and written communications are required to effectively convey information to both higher-level leadership and to subordinate enlisted sailors and marines.

## Military-Relevant Chemistry Research Prepares Officers for Fleet Readiness

Midshipmen execute research projects under tenured or tenure-track civilians, who account for the overwhelming majority of the Chemistry Department workforce, and permanent military professors, ensuring continuity of USNA’s mission of graduating warfighters that are ready to assume higher responsibilities in command, citizenship, and government. Students can conduct research in military-relevant areas, including fuels/rocket propellants,^20–25^ warfighter performance,^26^ chemical and biological warfare and health and readiness,^27–31^ group performance and warfighter preparation and leadership,^32,33^ Arctic security,^34^ materials science (e.g., coatings, underwater adhesives, etc.),^35–39^ and sensors.^40,41^ Midshipmen can also perform studies in more traditional areas of basic research (e.g., synthesis,^42,43^ biochemistry,^44,45^ and electrochemistry).^46^ Midshipmen work with faculty for 6 hours per week in research laboratories for at least one semester, although the majority of students opt to conduct a full year of research. Exceptional students are selected to participate in the Trident Scholar Research Program, which allows midshipmen to dedicate more time to independent research. The Chemistry Department typically has ∼2 Trident Scholars each year, and these students spend up to 12 hours per week working under one of the ∼25 research-active chemistry faculty. Our majors have a wide array of research projects at their disposal involving both fundamental and applied research in military-relevant areas, some of which are highlighted below. Here, they can further expand their technical competencies and master a wide range of complex instruments.

### Explosives and Fuels

Midshipmen study research questions with our faculty in the field of fuels and/or rocket propellants, including developing and understanding the physical properties of model mixture systems for synthetic and petroleum fuels^24^ and their effects on engine components,^21,25,47^ and the synthesis and development of polymeric binders for solid rocket fuels.^20^ Other efforts focus on the production of ultrahigh-purity hydrogen fuel for unmanned vehicles.^40^ In their work with military fuels and O-rings, midshipmen master the use of instruments that measure density, viscosity, surface tension, tensile strength, hardness, speed of sound, and flash point. With this knowledge, they can guide sailors who conduct fuel analyses aboard naval vessels.

### Materials

A large proportion of our faculty conduct basic and applied research in the development of military-relevant materials. Such efforts include the use of ionic liquids to transform biopolymers into materials that offer UV protection, have flame retardant properties, or shield from electromagnetic interference.^35–37^ USNA faculty and midshipmen are also invested in the development of bioinspired underwater adhesives for fleet applications and readiness.^38^ Here, midshipmen use and assess data from advanced instruments such as scanning electron and confocal microscopes and work with Navy-specific materials.

### Warfighter Protection

Midshipmen work with USNA faculty on research that provides warfighter protection against both chemical and biological weapons. Projects investigate the destruction of chemical warfare agents which help in designing personal protective gear^48^ and exploit bacterial and viral systems in order to develop novel drugs and protect the warfighter from diseases of infectious etiology.^27,28^ For example, students have synthesized metal–organic frameworks and assessed their systems using scanning electron microscopes, phosphorus-NMR, and thermal gravimetric analysis. Of note, biology and biochemistry research projects require building a strong foundation in biology in the absence of a life sciences degree program at USNA. Midshipmen work with faculty and use both standard (e.g., gel electrophoresis, protein purification, etc.) and more advanced biochemistry techniques (e.g., RT-qPCR and MALDI-MS). These research experiences in biology and biochemistry are instrumental in preparing midshipmen for medical school. A number of recent USNA Trident Scholars have worked in the laboratories of faculty conducting research in this area, highlighting both faculty and midshipmen commitment to tackling these increasingly imminent problems.^48–51^

### Publications

Midshipmen excellence in research and service has been recognized by coauthorship on peer-reviewed journal articles,^20–46^ recognition at national conferences, and even military achievement medals for their scientific contributions. Chemistry Department faculty publish ∼25–30 publications a year in a variety of peer-reviewed sources. Midshipmen research is also disseminated in nontraditional venues, including through the Defense Technical Information Center^48–59^ and in patents.^60^

### Midshipmen Presentations, Awards, and Prestigious Scholarships

Students regularly present their work at large conferences, including local and national ACS meetings. Midshipmen also attend the Inter-Academy Chemistry Symposium (IACS) every year, which is hosted at one of the military service academies on a rotational basis. These experiences allow midshipmen to engage with the broader scientific community to communicate important, military-relevant research. Some midshipmen research contributions have been recognized on the national and military level,^38^ and some students successfully compete in international scholarship programs, such as the Rhodes, Marshall, and Churchill Scholarships.

Advanced research and postgraduate opportunities are available to a subset of students. Some students seeking service assignments in the nuclear Navy successfully have competed to become Admiral Frank Bowman Scholars. This program requires midshipmen to complete a summer internship and conduct military-relevant research, and allows students to obtain a graduate degree at the Naval Postgraduate School (NPS, a separate institution that awards master’s and doctoral degrees) in a technical discipline prior to entering the fleet. Midshipman research projects at USNA prepare them for an NPS experience that focuses on Cognitive Readiness (maintaining the intellectual and technological edge fundamental to a decisive advantage at sea) and Intellectual Leadership (rapid development and adoption of new technologies, competencies, and strategic thinking needed to deter, fight, and win).^61^

## Collaborative Efforts Strengthen the Caliber and Impact of Chemistry Research

USNA chemistry faculty have embraced independent collaboration with researchers at academic institutions, government agencies, and the private sector (Figure 2) as a way to optimize research opportunities. Through collaboration, midshipmen strengthen their research experiences by engaging with scientists at large research institutions, industry, and government laboratories like those at Uniformed Services University (USU) and the U.S. Naval Research Laboratory (NRL). Many midshipmen also apply for summer internships, where they participate in an off-site research experience. These opportunities are intended to educate and inspire midshipmen by broadening their scholarship and leadership experiences, developing their critical thinking skills, and deepening their appreciation for practical applications of their academic studies.^9^ As future officers in the U.S. Navy and Marine Corps, midshipmen chemistry majors benefit from project-based summer internships, where they are able to bridge a gap between their chemistry course material and operational challenges while engaging in research projects that enable them to think critically and work to solve complex problems within the fleet. A recent chemistry graduate who traveled to Greenland for an internship noted, “Project-based research at USNA helped me create a mental framework for how I could apply my years of strong academic foundation to contribute towards solving real local, regional, and global problems, which was really inspiring as an undergraduate.”^62^

**Figure 2:**
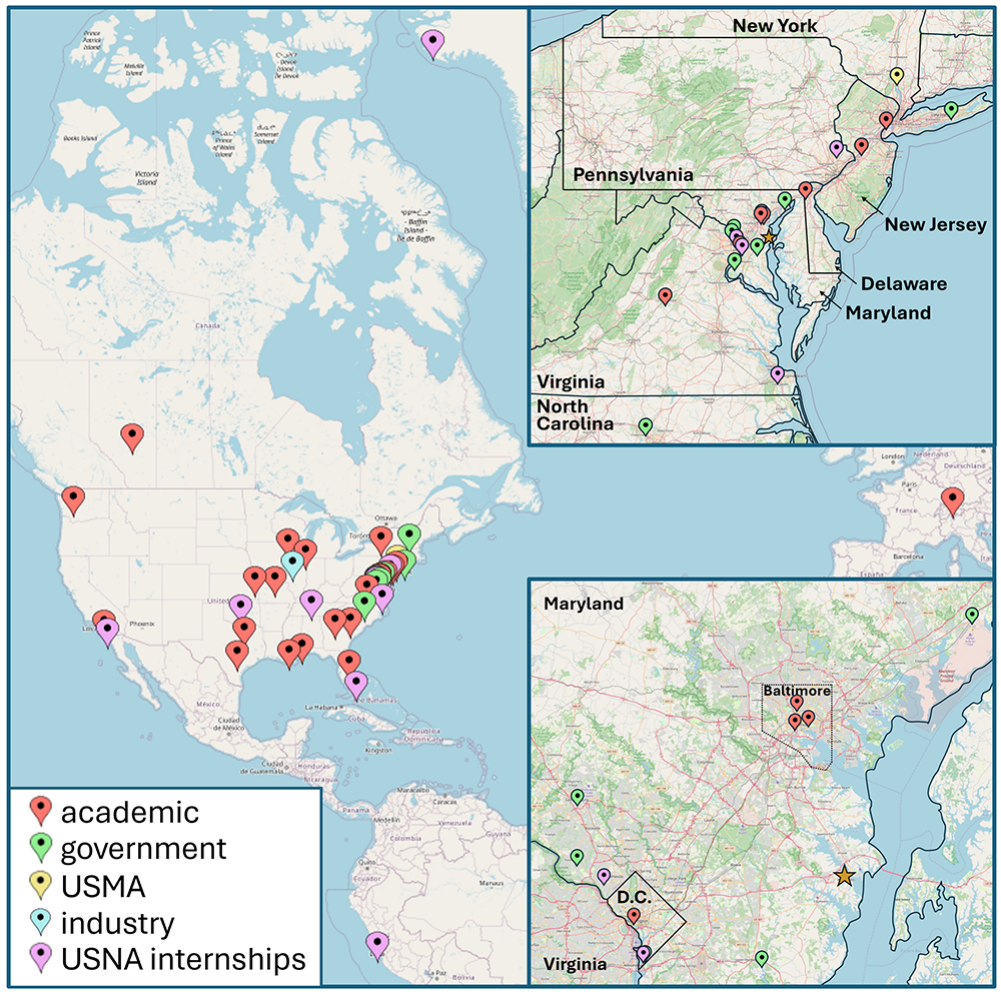
Locations of the USNA collaborations and internships. USMA designates the United States Military Academy at West Point.

### Funding for USNA Faculty and Midshipmen

USNA faculty are on 10 month contracts and must secure funding to pay for their summer salary, equipment, and supplies. While USNA faculty apply for funding via traditional avenues like the National Science Foundation (NSF), they are also funded by other entities such as the Defense Threat Reduction Agency (DTRA) and the Office of Naval Research (ONR). ONR also provides some general funding to USNA to support midshipmen conference travel, internships, and some research activities.

## Conclusion

The United States Naval Academy is in many ways similar to other undergraduate institutions, with a rigorous core curriculum, an ACS-accredited chemistry degree, and undergraduate research opportunities. However, USNA is distinct in its overarching focus on preparing graduates for warfighter professions with an emphasis on leadership, technical adaptability, and military applications throughout. USNA’s chemistry program graduates independent and confident thinkers who can adapt to challenges presented in military situations and who can lead sailors and marines under those conditions. While our context is unique, our model of utilizing collaboration and pursuing research programs specific to the institutional mission may be widely applicable to other undergraduate institutions.

## Supplementary Material



## Notes

Additional information about USNA’s “Plebe Chemistry” and “Integrated Laboratory” curricula (PDF)
