# Supplementary material for: Chemistry for the Warfighter: Midshipman Research at the U.S. Naval Academy
Source: ACS Omega. 2025 Dec 30;11(1):1–7. doi: 10.1021/acsomega.5c07144 (PMC12809761; doi:10.1021/acsomega.5c07144)
Supplement: Supplementary file 1 [file ao5c07144_si_001.pdf]

Supporting Information

Chemistry for the Warfighter: Midshipman Research at the U.S. Naval Academy

Leighanne A. Brammer Basta, Dianne J. Luning Prak, Melonie A. Teichert, and Elizabeth A. Yates*

AUTHOR ADDRESS: Department of Chemistry, United States Naval Academy, 572M Holloway Road, Annapolis, Maryland 21402, United States

*prak@usna.edu

Supporting information includes the following content: Additional information about Plebe Chemistry and the Integrated Laboratory program with representative naval applications.

Plebe Chemistry. Foundations of Chemistry I and II, commonly referred to as "Plebe Chemistry," is a two-semester general chemistry course sequence with both lecture and laboratory components required for all plebes (freshmen), regardless of their major. Class sizes are limited to 20 students, and faculty teach both the lecture and accompanying lab. For this reason, the USNA Chemistry Department is large, with ~35 tenure-track civilian faculty members, roughly five permanent or rotational military professors, and several adjunct instructors. Course content is presented via an atoms-first approach, and the USNA faculty employ a variety of evidence-based pedagogical practices. Plebe chemistry topics include those that are Navy-relevant, including water treatment and analysis (*e.g.* methods for purifying sea and fresh water), submarine air treatment (*e.g.* generation of O_2), corrosion, fuels and

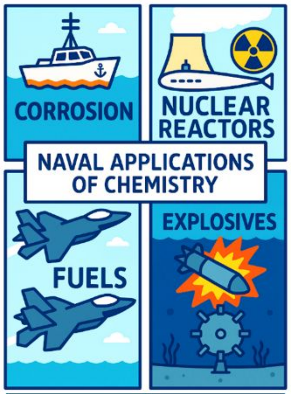

Example Lab Topic	Naval Application
Boiler water analysis	Water treatment
Radioactivity	Nuclear chemistry
Energy content of fuels	Fuels and explosives

Figure S1. *Representative naval applications of chemistry.*

lubricants, explosives, nuclear energy, as well as chemical and biological warfare, and representative plebe chemistry labs with a naval application emphasis are shown in Figure S1.

Integrated Lab Program. In each semester, midshipmen spend 6 hours per week in IL where they conduct experiments that investigate multiple areas of chemistry simultaneously, depending on the courses in which they are concurrently enrolled. This approach alleviates scheduling constraints typically associated with traditional courses with lab requirements and allows midshipmen the flexibility to conduct a full year of research with a faculty member their senior (1/C) year. Of note, midshipmen spend their summers immersed in Navy-relevant training all over the world and do not have the opportunity to conduct independent research at USNA during the summer months like their civilian counterparts.

Table S1. Representative lab topics in the Integrated Lab sequence that are Navy-relevant.

Example Lab Topic	Naval Application
Quantum dots	Nanomaterials for use in electronics and/or sensors, lubricants in motors, etc.
Polymer networks	Materials
Superconductors	Shielding of electronics in military reconnaissance and communications satellites, etc.

NOTES

Figure S1 was generated using Sora, ChatGPT (OpenAI). The author generated this graphic in part with GPT, OpenAI's large-scale language-generation model. Upon generating draft image, the author reviewed, edited, and revised the image to their own liking and takes ultimate responsibility for the content of this publication.
